# Antimicrobial Efficacy of Impregnated Human Acellular Dermal Substitutes in Burn Wound Models

**DOI:** 10.3390/antibiotics14070707

**Published:** 2025-07-14

**Authors:** Marianna Hajská, Elena Kurin, Silvia Bittner Fialová, Marian Vidiščák, Arpád Panyko

**Affiliations:** 1 4th Department of Surgery, Faculty of Medicine and University Hospital Bratislava, Comenius University Bratislava, Ružinovská 6, 82606 Bratislava, Slovakia; marianna.hajska@fmed.uniba.sk (M.H.); marian.vidiscak@fmed.uniba.sk (M.V.); arpad.panyko@fmed.uniba.sk (A.P.); 2 Department of Pharmacognosy and Botany, Faculty of Pharmacy, Comenius University Bratislava, Odbojárov 10, 83232 Bratislava, Slovakia; elena.kurin@uniba.sk

**Keywords:** wound infections, acellular dermal matrices, antimicrobial agents, *Pseudomonas aeruginosa*, *Acinetobacter baumannii*, 1% acetic acid

## Abstract

Burn wound infections remain a major clinical challenge due to delayed healing, scarring, and the risk of sepsis, especially when complicated by multidrug-resistant (MDR) Gram-negative pathogens and biofilm formation. Acellular dermal matrices (ADMs) are widely used in reconstructive and burn surgery, yet they lack intrinsic antimicrobial activity, necessitating their combination with topical agents. **Background/Objectives**: This study investigates the antimicrobial and cytocompatibility profiles of ADMs impregnated with various antimicrobial agents, using in vitro planktonic and biofilm burn wound models. While the incorporation of antimicrobials into scaffolds has been previously explored, this study is, to our knowledge, the first to directly compare seven clinically relevant antimicrobial agents after they were impregnated into an ADM in a standardized in vitro model. **Methods:** Seven topical antimicrobials were tested against MDR *Pseudomonas aeruginosa* and *Acinetobacter baumannii* from burn patients. **Results**: The ADM with 1% acetic acid (AA) showed superior antimicrobial activity, achieving > 7 log_10_ reductions in planktonic assays and complete inhibition of *P. aeruginosa* biofilms. In NIH 3T3 fibroblast cytotoxicity assays, the 1% AA ADM maintained cell viability at control levels, indicating excellent biocompatibility. Compared with agents such as Betadine^®^, Octenilin^®^, and colistin, which showed cytotoxicity, and Prontosan^®^, which showed low efficacy, 1% AA uniquely combined potent antibacterial effects with minimal toxicity. **Conclusions**: Among the seven antimicrobial agents impregnated into ADMs, 1% AA demonstrated a unique efficacy and safety profile, supporting its potential for clinical application in integrated wound dressings and implantable biomaterials for infection control in burn care.

## 1. Introduction

Acellular dermal matrices (ADMs) are widely utilized in burn and reconstructive surgery, as well as in plastic surgery and orthopedics, due to their ability to support tissue regeneration [1,2,3]. Additionally, ADMs have applications in the management of chronic wounds and in tissue reconstruction [4,5]. These dermal allografts, completely devoid of cellular components, serve as scaffolds for in vitro cell cultivation, including dermal fibroblasts and keratinocytes [6]. An ADM is derived from cadaveric allografts through various decellularization techniques, like physical, chemical, or biological, often used in combination to preserve the extracellular matrix structure while removing immunogenic elements [7]. Despite being non-toxic and non-antigenic, a standard ADM lacks inherent antimicrobial properties, necessitating the application of topical antimicrobial agents to reduce the risk of infection.

Burn wound infections represent a major complication in the acute phase of burn disease. Factors such as extensive wound contamination, burn-induced immunosuppression, prolonged hospitalization, and invasive procedures facilitate infections by multidrug-resistant nosocomial pathogens [8]. The most common isolated bacteria from burn wounds include *Pseudomonas aeruginosa*, *Staphylococcus aureus*, *Acinetobacter baumannii*, *Enterococcus faecalis*, *Escherichia coli*, *Klebsiella pneumoniae*, and *Enterobacter* spp. [9]. Infections significantly prolong wound healing, impair dermal maturation, and contribute to pathological scarring. Additionally, bacterial invasion of deeper tissues can lead to disseminated infection and sepsis [10,11].

A key challenge in burn wound management is the increasing prevalence of antimicrobial resistance, driven in part by biofilm formation. Bacterial biofilms consist of structured microbial communities encased in an extracellular polymeric substance, which enhances resistance to antibiotics, disinfectants, and host immune responses [12]. Biofilms are commonly detected in burn wounds, further complicating treatment [13].

Scientific evidence suggests that the application of topical antimicrobial agents can significantly reduce morbidity and mortality in burn patients by preventing and controlling wound infections [10]. These agents can be applied at all stages of wound management, providing both prophylactic and therapeutic benefits. Importantly, systemic antibiotic therapy often fails in burn wounds due to compromised vascularization, further emphasizing the necessity of localized antimicrobial interventions [11]. Topical antimicrobial agents are widely recommended in clinical practice guidelines for burn wound care, primarily to prevent infections that can lead to delayed healing, sepsis, or death. Notably, some topical preparations remain a commonly used agent in deeper burns and low-resource settings due to their broad-spectrum activity and low resistance profile, particularly against *Staphylococcus aureus* and *Pseudomonas aeruginosa*. In contrast, the use of topical antibiotics is typically limited to superficial or facial wounds, as evidence indicates that topical antibiotic application may contribute more significantly to antimicrobial resistance [14]. Therefore, in the context of rising antimicrobial resistance, selecting effective topical agents while minimizing the risk of resistance transmission remains a critical priority [15].

The aim of this study was to enhance the antimicrobial properties of ADMs by impregnating them with selected antimicrobial agents and evaluating its efficacy in in vitro burn wound models contaminated with multidrug-resistant *Pseudomonas aeruginosa* and *Acinetobacter baumannii*.

## 2. Results

### 2.1. Antibacterial and Antibiofilm Activity of Antiseptic Agents Impregnated on ADMs

**Figure 1:**
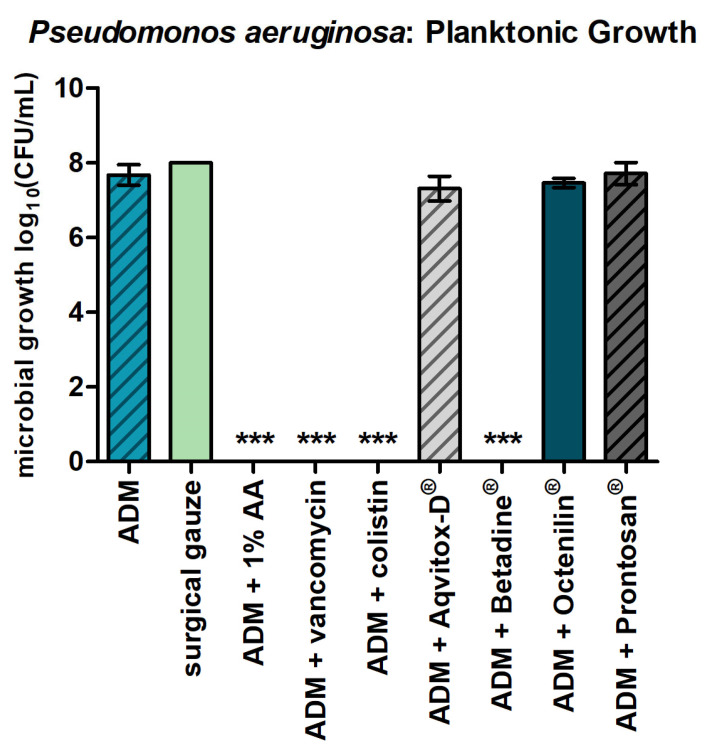
*P. aeruginosa* in planktonic form and ADMs with the tested agents. The bars represent the mean ± SD, n = 3. *** *p* < 0.001 for comparisons among the samples (ANOVA/Turkey).

The ADMs impregnated with 1% AA, vancomycin, colistin, and Betadine^®^ were significantly effective against the growth of the planktonic form of *P. aeruginosa*, as shown in Figure 1.

These treatments resulted in a log_10_ reduction of >7 CFU/mL in bacterial counts, indicating a strong inhibitory effect according to internationally recognized standards (Table 1). This substantial reduction reflects a marked decrease in viable bacterial cells compared with the untreated control, indicating a powerful inhibitory effect. According to standardized interpretation criteria, a reduction greater than 3 log_10_ CFU/mL is classified as strong antimicrobial activity, further underscoring the effectiveness of these treatments in suppressing bacterial growth. In contrast, ADMs treated with Aqvitox-D^®^, Octenilin^®^, and Prontosan^®^ showed no measurable antimicrobial activity.

Based on the standard interpretation, a reduction of >3 log_10_ was classified as strong antibacterial activity, 1–3 log_10_ as significant antibacterial activity, 0.5–1 log_10_ as slight antibacterial activity, and <0.5 log_10_ as no activity.

**Figure 2:**
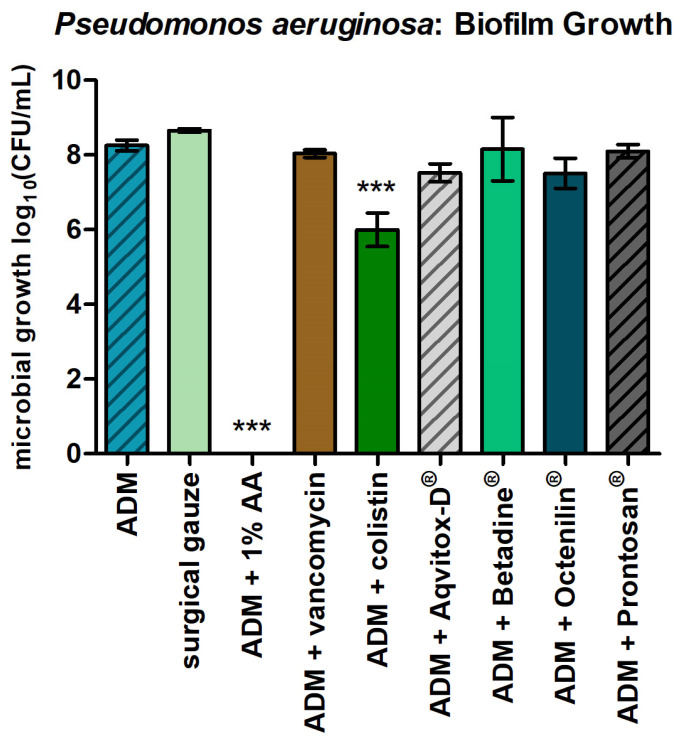
*P. aeruginosa* in a 24 h biofilm and the ADMs with the tested agents. The bars represent the mean ± SD, n = 3. *** *p* < 0.001 for comparisons among the samples (ANOVA/Turkey).

In the 24 h biofilm model of *P. aeruginosa*, only the ADM impregnated with 1% AA completely inhibited the biofilm growth (Figure 2), achieving a log_10_ reduction of >7, which qualifies as strong antimicrobial activity (Table 1). The ADM impregnated with colistin also significantly reduced biofilm formation, with a log_10_ reduction of 2.26, corresponding to significant antimicrobial activity. In contrast, the remaining agents showed no measurable effect on biofilm growth, with slight or no log reduction observed.

**Figure 3:**
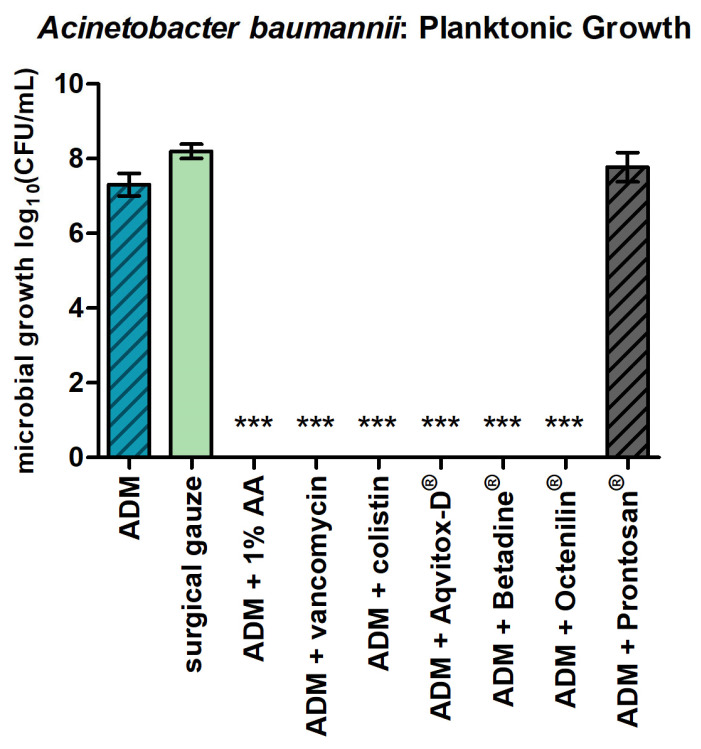
*A. baumannii* in planktonic form and the ADMs with the tested agents. The bars represent the mean ± SD, n = 3. *** *p* < 0.001 for comparisons among the samples (ANOVA/Turkey).

For *A. baumannii* in planktonic form (Figure 3), the ADMs impregnated with all tested agents, except Prontosan^®^, resulted in a significant reduction in bacterial growth. The overall log_10_ reduction reached >7, indicating a strong antimicrobial effect according to the standardized classification (Table 1).

**Figure 4:**
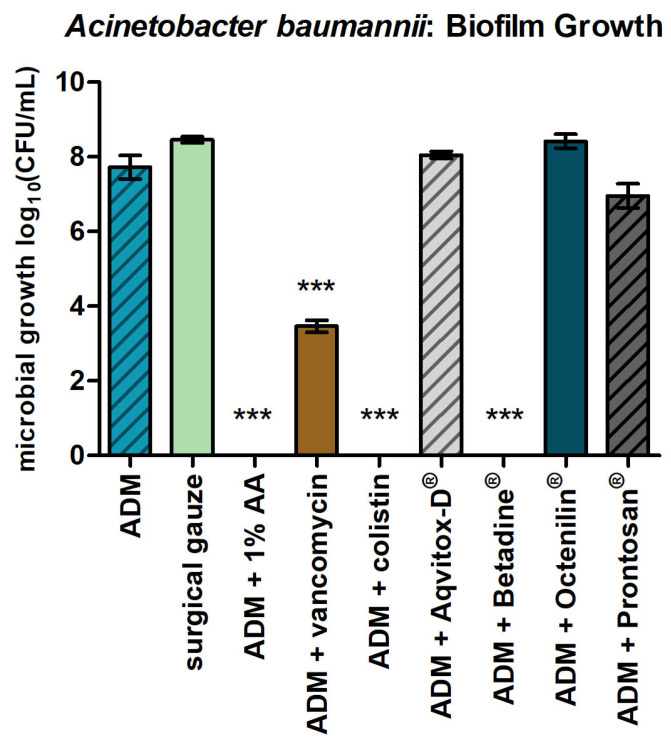
*A. baumannii* in a 24 h biofilm and the ADMs with the tested agents. The bars represent the mean ± SD, n = 3. *** *p* < 0.001 for comparisons among the samples (ANOVA/Turkey).

In the 24 h biofilm model of *A. baumannii* (Figure 4), the ADMs impregnated with 1% AA, Betadine^®^, colistin, and vancomycin demonstrated strong antimicrobial activity, with log_10_ reductions of >7 for 1% AA, colistin, and Betadine^®^ and 4.26 for vancomycin, all classified as strong effects according to Table 1. Prontosan^®^ showed slight antibacterial activity, with a log_10_ reduction of 0.76. In contrast, the ADMs treated with Aqvitox-D^®^ and Octenilin^®^ showed no measurable antimicrobial effect, indicating a lack of efficacy against biofilm growth.

### 2.2. Cell Viability: Cytotoxicity of Antimicrobial Agents Combined with ADMs

**Figure 5:**
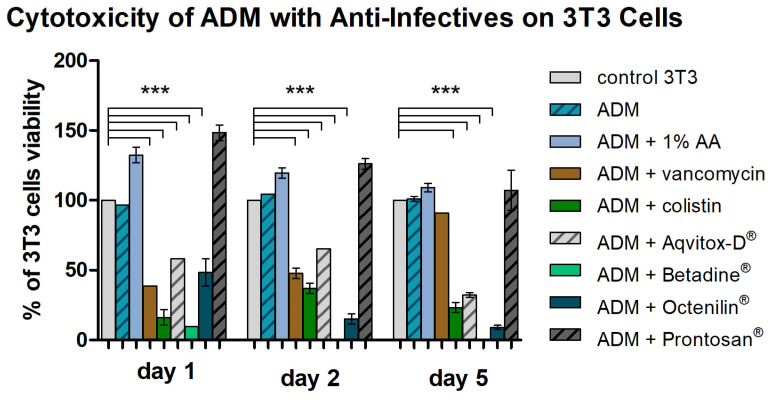
Evaluation of the cytotoxic effects of ADMs combined with various antibacterial agents on 3T3 fibroblasts, based on cell viability testing. Cell viability was assessed on days 1, 2, and 5 to capture both early and late responses. Viable adherent cells were detached by trypsinization and counted using a Bürker chamber; values are expressed as the percentage relative to untreated controls. The results represent the mean ±SD from three independent experiments (n = 3). *** *p* < 0.001 for comparisons among the samples (ANOVA/Tukey).

The cytotoxic effects of various antibacterial agents in combination with an acellular dermal matrix (ADM) were evaluated on 3T3 fibroblast cells over a 5-day period (Figure 5). Cell viability is expressed as the percentage relative to untreated 3T3 control cells.

On the first day, both the ADM alone and ADMs combined with 1% AA and Prontosan^®^ maintained high levels of cell viability, with significant increases observed comparable with or exceeding the control. These conditions showed no signs of cytotoxicity and maintained cell viability comparable with the control. The ADMs combined with vancomycin, Aqvitox-D^®^, or Octenilin^®^ showed moderate cytotoxicity (viability below 70%), while ADMs combined with colistin and, especially, Betadine^®^ demonstrated high cytotoxicity (viability below 30%) and reduced viability of fibroblasts, with all reductions being statistically significant.

On day two, the ADM combined with 1% AA and the ADM combined with Prontosan^®^ showed the lowest cytotoxicity, maintaining cell viability above normal levels, with a statistically significant increase. Next, the ADM combined with vancomycin and the ADM combined with Aqvitox-D^®^ demonstrated statistically significant moderate cytotoxicity, with cell viability dropping below 70%. Similarly, the ADM combined with colistin also showed statistically significant moderate cytotoxicity, with a viability around 40%. The ADMs combined with Octenilin^®^ and Betadine^®^ exhibited high cytotoxicity (viability below 30%), with Betadine^®^ showing the most pronounced effect, reducing cell viability to nearly 0%.

On day five, the highest cell viability was observed in treatments with ADMs combined with 1% AA and Prontosan^®^, both maintaining levels above the control, indicating no cytotoxicity. Notably, the ADM combined with vancomycin showed improved cell viability, reaching approximately 90%, suggesting a possible delayed protective or adaptive response. In contrast, the ADMs combined with colistin and Aqvitox-D^®^ exhibited statistically significantly high cytotoxicity, with cell viability dropping below 30%. Similarly, the ADMs combined with Octenilin^®^ and Betadine^®^ also demonstrated statistically significant severe cytotoxic effects, with Betadine^®^ reducing viability to nearly 0%.

Overall, the ADM in combination with 1% AA consistently demonstrated the most favorable biocompatibility, maintaining or even enhancing 3T3 cell viability at all observed time points, with levels exceeding the control baseline. In contrast, combinations with Betadine^®^, Octenilin^®^, colistin, and Aqvitox-D^®^ exhibited marked and, in most cases, statistically significant cytotoxicity, particularly after prolonged exposure, with Betadine^®^ showing the most severe effect.

## 3. Discussion

Infectious complications of burn wounds represent a significant medical challenge, contributing to delayed healing, the formation of deep burn scars, and, in cases where microorganisms infiltrate the systemic circulation, life-threatening conditions such as sepsis [10,11]. One of the major factors complicating the treatment of burn wound infections is the increasing bacterial resistance to antimicrobial agents. A key mechanism of this resistance is the formation of microbial biofilms [11].

ADMs are widely utilized across various surgical specialties, with their most common application in burn surgery. However, in clinical practice, it is currently employed only in combination with topically applied antimicrobial agents. Scientific studies have demonstrated that the use of topical antimicrobial agents reduces morbidity and mortality in patients with extensive burns [10]. A recent multicenter study reported excellent clinical outcomes with the use of an antimicrobial-coated acellular porcine dermal matrix in hernia repair [16]. Additionally, Yang et al. [17] demonstrated the beneficial effect of impregnated acellular dermal matrices (ADMs) in infection treatment by developing a levofloxacin-loaded porcine acellular dermal matrix hydrogel for urinary infection management.

In this study, we investigated the antimicrobial efficacy of impregnated acellular dermal replacements in in vitro models of burn wounds contaminated or infected with multidrug-resistant Gram-negative bacteria, which are among the most frequently isolated pathogens in such wounds. Unlike previous studies available in the literature [9,18], our work focused on ADMs impregnated with antimicrobial agents rather than a combination of ADMs with topically applied agents. According to the current literature, the concept of impregnated ADMs has not yet been introduced into clinical practice, nor has their efficacy against bacterial strains been studied in in vitro models.

Using a modified methodology based on Hammond et al. [19], we developed the following two in vitro burn wound models: a planktonic growth model and a biofilm model. We tested two multidrug-resistant Gram-negative bacterial strains, *P. aeruginosa* and *A. baumannii*, both isolated from burn patients. Seven topical antimicrobial agents were evaluated, as follows: 1% AA, vancomycin, colistin, Aqvitox-D^®^ (hypochlorous acid and sodium hypochlorite), Betadine^®^ (povidone-iodine), Octenilin^®^ (octenidine dihydrochloride), and Prontosan^®^ (polyhexamethylene biguanide hydrochloride). ADMs were prepared following the method described by Dragúňová et al. [6], impregnated with the respective antimicrobial substances, and subsequently applied to the in vitro burn wound models.

Our findings demonstrate that the ADM impregnated with 1% AA exhibited exceptional antimicrobial efficacy against the tested multidrug-resistant Gram-negative bacterial strains. This agent showed the strongest inhibitory effect against both planktonic bacteria and biofilm formation after 24 h. The strong antibacterial performance of 1% AA, characterized by log_10_ reductions exceeding > 7 in all planktonic assays, clearly surpasses the threshold for “strong” antimicrobial activity as defined by standard classification criteria. Moreover, 1% AA was the only agent that completely inhibited biofilm formation of *P. aeruginosa* and was one of the most effective agents against biofilm-forming *A. baumannii*, alongside colistin and Betadine^®^.

This is especially notable given the high tolerance of biofilms to conventional antibiotics and antiseptics [20,21]. AA, a weak organic acid, has historically been used in medicine; for example, Hippocrates recommended it for wound care. It is present in vinegar at a 3–5% concentration. It can be produced inexpensively via fermentation or synthesis, is stable upon autoclaving, and has favorable storage properties [22]. Moreover, AA biodegrades into CO_2_ and H_2_O, making it environmentally friendly and sustainable. Unlike antibiotics, it does not pollute soil and water or contribute to antimicrobial resistance. The antibacterial effect of AA is primarily attributed to its ability to penetrate bacterial cell membranes in its protonated form, leading to cytoplasmic acidification and disruption of vital cellular functions. This uncoupling effect dissipates the proton-motive force and inhibits bacterial respiration, ultimately compromising cell viability. Such a mechanism is particularly effective against planktonic bacterial forms [23]. Moreover, by lowering the wound bed pH, which is typically elevated in chronic wounds [24], AA can inhibit bacterial proliferation, suppress biofilm formation, and modulate the local environment to favor tissue regeneration. This acidification not only reduces the toxicity of bacterial by-products like ammonia but also helps restore physiological pH levels that support collagen synthesis, angiogenesis, and immune cell function, further enhancing its therapeutic potential [25]. Acidification of the wound environment, particularly in burns and chronic wounds, can reduce elevated alkaline pH levels toward a more physiological range, thereby supporting keratinocyte proliferation and tissue repair. It was shown that maintaining a wound pH of 4 significantly enhanced re-epithelialization, collagen synthesis, and overall healing compared with more neutral conditions [26]. Similarly, it was demonstrated that burn wounds treated with a pH 3.5 buffer exhibited significantly faster epithelialization than those treated with neutral or alkaline solutions [27]. Notably, this concentration of acetic acid has been used in our clinical practice for an extended period, particularly for the management of burn wounds, donor sites, and grafted areas, with very good local tolerance and without reports of irritation, supporting its potential applicability.

While other agents such as vancomycin and colistin showed similar antimicrobial potency in the planktonic phase, they failed to match the broad-spectrum and biofilm-targeting capacity of 1% AA. The fact that some commonly used antiseptics (e.g., Octenilin^®^, Aqvitox-D^®^, and Prontosan^®^) lacked significant efficacy further underscores the therapeutic potential of 1% AA in wound care and implant-associated infections. Cytotoxicity testing on NIH 3T3 fibroblast cells revealed substantial differences in the biocompatibility of the tested combinations. Once again, the ADM combined with 1% AA demonstrated outstanding results, consistently maintaining cell viability above 100% at all observed time points. These values not only reflect non-cytotoxic behavior but also confirm the biocompatibility of the material with fibroblasts. This may be partly attributed to the acidifying effect of AA, which helps restore the naturally acidic pH of intact skin, maintained by organic acid secretion from keratinocytes. In contrast, chronic wounds become alkaline, which promotes protease activity and the accumulation of toxic by-products, impairing the healing process [28]. Similarly, the ADM combined with Prontosan^®^ showed a non-toxic profile with a high cell viability comparable to the control; however, its antimicrobial activity was markedly limited, reducing its overall therapeutic value in infected-wound settings.

In contrast, Betadine^®^ and Octenilin^®^ showed severe and persistent cytotoxic effects, with Betadine^®^ reducing cell viability to nearly zero across all time points. This may be attributed to povidone-iodine’s ability to significantly reduce fibroblast proliferation, increase apoptosis and necrosis, and impair wound closure during early healing stages [29]. Although octenidine is considered safe for superficial use, its application in deeper wounds has been associated with tissue necrosis, chronic inflammation, and delayed healing, which may explain the observed in vitro cytotoxicity and suggests limited suitability for tissue-integrated therapies [30].

Similarly, Aqvitox-D^®^ led to a progressive decline in cell viability, likely due to the concentration-dependent cytotoxicity of hypochlorite, which at ≥0.01% impairs fibroblast adhesion and mitochondrial function. In addition, even diluted NaOCl solutions (0.025–0.0025%) have been shown to cause sublethal cellular damage [31]. Likewise, colistin led to a progressive decline in fibroblast cell viability, confirming its high cytotoxic potential, particularly with prolonged exposure; this is consistent with its known neurotoxicity in clinical use, which results in oxidative stress and apoptosis [32]. Additionally, colistin is nephrotoxic, inducing apoptosis and autophagy, leading to colistin-induced nephrotoxicity [33]. Even vancomycin, though showing recovery on day 5, initially exhibited moderate cytotoxicity. It has been shown to affect fibroblasts at higher concentrations, indicating a dose-dependent cytotoxic response [34].

While the direct comparison of these specific agents, particularly the focus on 1% AA, is novel, it should be noted that the broader concept of loading antimicrobial agents into scaffolds is not entirely new. However, to our knowledge, this is the first study to directly compare seven different antimicrobial agents post-impregnation in a standardized in vitro ADM model, thereby providing comparative insights into their respective antimicrobial, antibiofilm, and cytocompatibility profiles. This approach revealed the distinct advantages of 1% AA. From a clinical standpoint, the combination of robust antimicrobial and antibiofilm activity and excellent cytocompatibility makes 1% AA an ideal candidate for use in tissue-integrated wound dressings or implant coatings. This dual advantage is critical in managing infected wounds where both bacterial clearance and tissue regeneration are essential, making it especially promising for applications in regenerative medicine and chronic wound management.

## 4. Materials and Methods

### 4.1. Acellular Allodermis—Acellular Dermal Matrix (ADM)

A human ADM (Figure S1) was prepared using a novel decellularization method developed by Dragúňová [6]. The process involved enzymatic separation of the epidermis from cadaveric skin allografts using a trypsin (Sigma-Aldrich, Merck KGaA, Darmstadt, Germany) and ethylenediaminetetraacetic acid (EDTA) solution (Sigma-Aldrich, Merck KGaA, Darmstadt, Germany), followed by mechanical removal and dermal lavage in hypotonic injection water to induce cell lysis. Histological analysis and cytotoxicity assays confirmed the complete removal of cellular components and the non-toxicity of the final product. The decellularized ADM was subsequently cryopreserved in the Cell and Tissue Bank. Before experimentation, the ADM was meshed at a 3:1 ratio, cut into 5 × 5 cm pieces, and impregnated for 24 h in antimicrobial solutions (1 mL), with each 25 cm^2^ piece submerged in 100 mL of the respective solution.

### 4.2. Bacterial Strains

Two multidrug-resistant Gram-negative bacterial strains, *Pseudomonas aeruginosa* and *Acinetobacter baumannii*, were selected for testing. These strains were isolated from burn patients hospitalized in the Burn ICU and characterized by their resistance profiles at the University Microbiology Institute, CU Bratislava.

### 4.3. Antimicrobial Agents

Seven antimicrobial agents, including five topical antimicrobials and two antibiotics, were used for the ADM impregnation (1 mL for each), as follows: 1% AA solution (Sigma-Aldrich, Merck KGaA, Darmstadt, Germany); Aqvitox-D^®^ = mixture of hypochlorous acid and sodium hypochlorite < 0.05% (Aqvitox Technology s.r.o., Bratislava, Slovakia); Betadine^®^ solution = 10% povidone-iodine (EGIS Pharmaceuticals PLC, Budapest, Hungary); Octenilin^®^ solution = octenidine dihydrochloride (Schülke & Mayr GmbH, Vienna, Austria); Prontosan^®^ solution = 0.1% polyhexamethylene biguanide hydrochloride (B.Braun Konzern Medical, Melsungen, Germany); 0.3 mg colistin (1 MIU in 100 mL physiological saline, KOLOMYCÍN 1 MIU TEVA Pharmaceuticals, Tel Aviv, Israel); and 50 mg vancomycin (1 MIU in 20 mL physiological saline, EDICIN 1 g, Sandoz Group AG, Basel, Switzerland). The comparison of antibacterial agents in this study was based on clinically relevant doses corresponding to commonly used concentrations in practice rather than on equipotent antimicrobial units. Control samples consisted of the ADM immersed in physiological saline (B.Braun Konzern Medical, Melsungen, Germany) and sterile gauze (5 × 5 cm, Sterilux^®^ ES, Heidenheim an der Brenz, Germany).

### 4.4. In Vitro Burn Wound Models

Two distinct in vitro burn wound models were created based on a modified method by Hammond et al. [19], as follows:

Model A—simulated planktonic bacterial growth:

For model preparation [9,19], sterile cellulose disks (6 mm in diameter, Thermo Scientific™ Oxoid™ Disc, Waltham, MA, USA) were placed onto Luria-Bertani agar (LBA, Sigma-Aldrich, Merck KGaA, Darmstadt, Germany) plates. A bacterial suspension containing 10^2^ CFU was applied to each disk. The impregnated ADM (1 mL of antimicrobial agent), non-impregnated ADM, or sterile gauze (5 × 5 cm, Sterilux^®^ ES, Heidenheim an der Brenz, Germany) was placed over the contaminated disks immediately after inoculation to simulate planktonic bacterial growth. To ensure uniform contact between the ADM and bacterial disks, sterile Petri dishes (60 mm in diameter) were placed over the ADM surface.

Model B—simulated biofilm formation:

Sterile cellulose disks were prepared as in Model A. However, the disks were incubated for 24 h at 37 °C to promote biofilm formation prior to ADM application. Before placing the ADM, the disks were gently rinsed with sterile phosphate-buffered saline (PBS, Sigma-Aldrich, Merck KGaA, Darmstadt, Germany) to remove non-adherent planktonic bacteria, leaving only the biofilm intact. The impregnated ADM, non-impregnated ADM, or sterile gauze was then placed on top of the disks. To ensure uniform contact between the ADM and bacterial disks, sterile Petri dishes (60 mm in diameter) were placed on top of the ADM surface.

For both models, following 24 h of incubation at 37 °C, each cellulose disk was transferred into a sterile test tube containing 1 mL of PBS. Bacteria were released by vigorous shaking at 2200 rpm for three consecutive cycles of two minutes each. The bacterial suspension was serially diluted in PBS using decimal dilutions, and 10 µL from each dilution was plated onto LBA plates. The plates were incubated for 20 h at 37 °C, after which bacterial colonies were enumerated and calculated as the CFU per disk to assess the antimicrobial efficacy.

### 4.5. Determination of Microbial Growth Reduction

The antimicrobial efficacy was quantified as the difference in log_10_ colony-forming units per milliliter (Δlog_10_ CFU/mL) between the untreated controls and treated samples (Table 1). The number of viable microorganisms in the samples was assessed by calculating both the colony-forming units per milliliter (CFU/mL) and the total microbial count (expressed as CFU). To evaluate the antimicrobial efficacy, the reduction in microbial growth was determined by comparing the microbial load after 24 h of incubation to the initial value, in accordance with Wiegand et al. [35]. The antimicrobial efficacy was indicated by a reduction in bacterial counts in log_10_ CFU/mL compared with the baseline control and classified according to internationally recognized standards. The reduction in growth was calculated using Equation (1), as follows:

ΔlogCFU/mL = log_10_(CFUc/mL) − log_10_(CFUs/mL) (1)

where CFUc is the control, and CFU is the sample.

The extent of antimicrobial activity was interpreted based on the magnitude of the log reduction, as in Table 2.

The limit of detection (LOD) for the plating method was 1 CFU/mL (log_10_ = 0), meaning that counts below this value could not be reliably detected. For these samples, reductions greater than 7 log_10_ are reported as >7 log_10_ to reflect the maximum detectable reduction based on the LOD.

### 4.6. Cytotoxicity Tests

3T3 NIH murine fibroblasts (3T3) were purchased from the ECCACC collection (Lambda Life, Petržalka, Slovakia, ECCACC number: 850 22,108) and cultivated in Dulbecco-modified medium (Pan Biotech, Aidenbach, Germany) supplemented with 10% fetal calf serum (Pan Biotech, Aidenbach, Germany). The 3T3 cells were routinely cultured according to standard procedures (incubated in humidified air at 37 °C with 7.0% CO_2_) and passaged at least once a week.

Cytotoxicity was assessed using a modified method based on [36]. The murine fibroblast cell line NIH 3T3 was cultured as described above until reaching confluence. At time 0 h, 60,000 cells were seeded into 6 cm diameter Petri dishes, followed by incubation at 37 °C in a humidified atmosphere with 7% CO_2_. Three experimental groups were established, as follows:

Untreated 3T3 cells (negative control);

3T3 cells with acellular dermal matrix;

3T3 cells with ADM impregnated with 100 μL of one of the tested antibacterial agents, including 1% AA, Aqvitox-D^®^, Betadine^®^, Octenilin^®^, Prontosan^®^, colistin, and vancomycin.

Each sample was tested in triplicate. The antibacterial agents were applied by directly placing ADM fragments pre-impregnated with the respective agent onto the 3T3 monolayers. Cell morphology was monitored microscopically before and after treatment. After 24 h (Figures S2 and S3), 48 h, and 5 days, the ADM was removed, and the wells were washed with PBS and EDTA. Cells were detached using trypsin and counted using a Bürker’s counting chamber. The number of adherent cells at each time point was compared with the untreated control. The percentage of viable, adherent cells was calculated to reflect the cytotoxicity, with lower percentages indicating higher cytotoxic effects. All procedures were performed under sterile conditions.

### 4.7. Statistical Analysis

All measurements were performed in triplicate (n = 3), and the results are expressed as the mean ± standard deviation (SD). The statistical analysis was carried out using one-way ANOVA, followed by Tukey’s post hoc test for multiple comparisons. Analyses were conducted using GraphPad Prism version 5.01 software (GraphPad Software, Boston, MA, USA). Differences were considered statistically significant at the following thresholds: *p* < 0.05 (*), *p* < 0.01 (**), and *p* < 0.001 (***).

## 5. Conclusions

This study highlights the potential of ADM impregnated with antimicrobial agents as a promising approach for the treatment of burn wound infections, particularly those caused by multidrug-resistant Gram-negative bacteria. Our in vitro models demonstrated that the ADM impregnated with 1% AA exhibited superior antimicrobial activity against both planktonic and biofilm-associated forms of Pseudomonas aeruginosa and Acinetobacter baumannii. Among all tested agents, 1% AA was the only compound capable of completely inhibiting biofilm formation by *P. aeruginosa*, while also showing strong efficacy against *A. baumannii*. These results position AA as a highly effective antimicrobial agent with robust antibiofilm properties. In addition to its antimicrobial efficacy, 1% AA demonstrated excellent biocompatibility, maintaining high fibroblast viability throughout the observation period. This contrasts with the cytotoxic profiles observed for several commonly used antiseptics and antibiotics, such as Betadine^®^, Octenilin^®^, and colistin. The combination of strong antibacterial effect and cytocompatibility is particularly important for applications where both infection control and tissue regeneration are essential. Given its low cost, environmental safety, and lack of contribution to antimicrobial resistance, AA represents a sustainable and clinically relevant solution for infected-wound management. While the concept of loading antimicrobial agents into scaffolds is not entirely new, our study is, to our knowledge, the first to directly compare seven different antimicrobial agents post-impregnation into an ADM using a standardized in vitro model. This allowed us to identify the unique performance of 1% acetic acid, particularly its ability to inhibit biofilm formation by *P. aeruginosa*, while maintaining high cytocompatibility. The use of an impregnated ADM, rather than topical application alone, offers the advantage of prolonged local release, enhanced tissue integration, and potential use in implantable devices. Further preclinical and clinical studies are warranted to validate these findings and to explore the translational potential of AA-based ADM dressings in burn care and regenerative medicine.

## Figures and Tables

**Table 1:** The bacterial growth-inhibitory activity expressed as log_10_ colony-forming units per milliliter (Δlog_10_ CFU/mL) of the various agents impregnated on an ADM against the planktonic form and biofilms of *P. aeruginosa* and *A. baumannii*. The limit of detection (LOD) for the plating method was 1 CFU/mL, meaning that counting below this value could not be reliably detected. For these samples, a value of >7 log_10_ is reported as the maximum detectable reduction based on the LOD.

	Δlog_10_(CFU/mL)						
	1% AA	Vancomycin	Colistin	Aqvitox-D^®^	Betadine^®^	Octenilin^®^	Prontosan^®^
*Pseudomonas aeruginosa*: Planktonic Growth	>7	>7	>7	0.37	>7	0.22	−0.04
antimicrobial activity	strong	strong	strong	no	strong	no	no
*Pseudomonas aeruginosa*: Biofilm Growth	>7	0.22	2.26	0.74	0.10	0.75	0.16
antimicrobial activity	strong	no	significant	slight	no	slight	no
*Acinetobacter baumannii*: Planktonic Growth	>7	>7	>7	>7	>7	>7	−0.47
antimicrobial activity	strong	strong	strong	strong	strong	strong	no
*Acinetobacter baumannii*: Biofilm Growth	>7	4.26	>7	−0.33	>7	−0.70	0.76
antimicrobial activity	strong	strong	strong	no	strong	no	slight

**Table 2:** The interpretation of antibacterial activity.

<0.5-log growth reduction	no antimicrobial activity
0.5–1.0-log growth reduction	slight antimicrobial activity
>1.0–3.0-log growth reduction	significant antimicrobial activity
>3.0-log growth reduction	strong antimicrobial activity

## Data Availability

All data generated or analyzed during this study are included in the article.

## Supplementary Materials

The following supporting information can be downloaded at: https://www.mdpi.com/article/10.3390/antibiotics14070707/s1, Figure S1: Histological image of acellular dermal matrix (ADM) showing preserved extracellular matrix and absence of cells, confirming scaffold integrity; Figure S2: Morphology of NIH 3T3 fibroblasts after 24-h incubation with (A) sterile gauze (control) and (B) with acellular dermal matrix (ADM) without antibacterial agents; Figure S3: Morphology of NIH 3T3 fibroblasts after 24-h incubation with 1% acetic acid (A) and with Betadine^®^ (B).

## Abbreviations

The following abbreviations are used in this manuscript:

AA	Acetic Acid
ADMs	Acellular Dermal Matrices
CFU	Colony Forming Units
EDTA	Ethylenediaminetetraacetic Acid
NIH/3T3	Mouse Embryo Fibroblast Cell Line Clone 3T3
PBS	Phosphate-Buffered Saline
