# Supplementary material for: Antimicrobial Efficacy of Impregnated Human Acellular Dermal Substitutes in Burn Wound Models
Source: Antibiotics (Basel). 2025 Jul 14;14(7):707. doi: 10.3390/antibiotics14070707 (PMC12291905; doi:10.3390/antibiotics14070707)
Supplement: Supplementary file 1 [file antibiotics-14-00707-s001.zip › antibiotics-3724312-supplementary.pdf]

Supporting Information

Antimicrobial Efficacy of Impregnated Human Acellular Dermal Substitutes in Burn Wound Models

Marianna Hajská [1,†], Elena Kurin [2,†], Silvia Bittner Fialová [2,*], Marian Vidiščák [1], Arpád Panyko [1]

[1] 4th Department of Surgery, Faculty of Medicine and University Hospital Bratislava, Comenius University Bratislava, Ružinovská 6, 82606 Bratislava, Slovakia; e-mail: marianna.hajska@fmed.uniba.sk, marian.vidiscak@fmed.uniba.sk, arpad.panyko@fmed.uniba.sk

[2] Department of Pharmacognosy and Botany, Faculty of Pharmacy, Comenius University Bratislava, Odbojárov 10, 83232 Bratislava, Slovakia; e-mail: fialova@fpharm.uniba.sk, elena.kurin@uniba.sk

* Correspondence: fialova@fpharm.uniba.sk; Tel.: +421-2-9016-9206

† These authors contributed equally to this work.

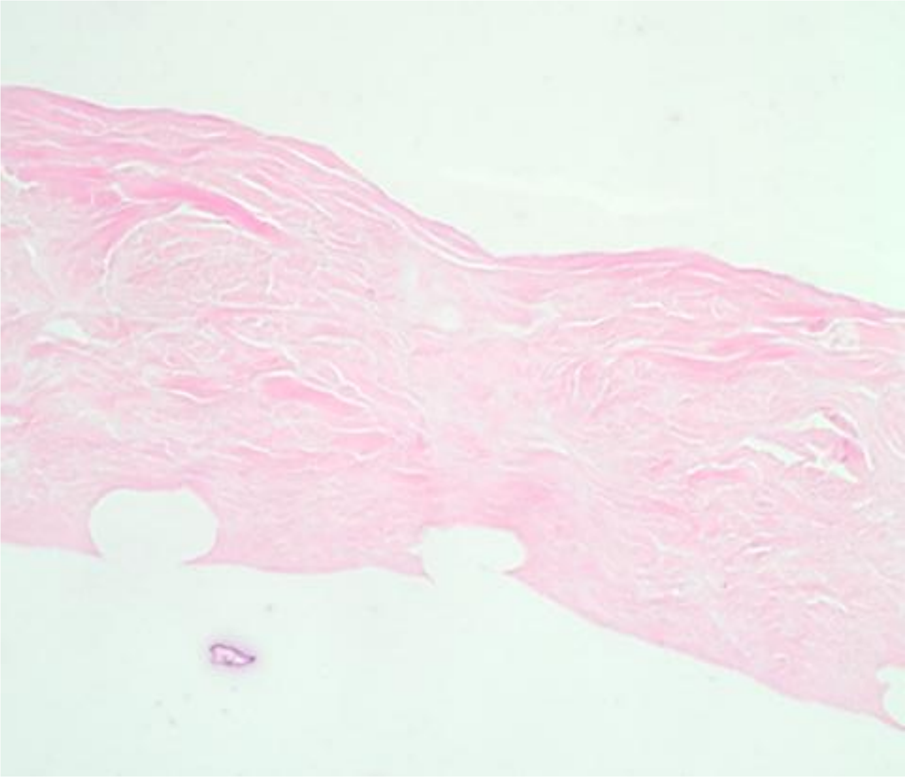

Figure S1 Histological image of acellular dermal matrix (ADM) showing preserved extracellular matrix and absence of cells, confirming scaffold integrity.

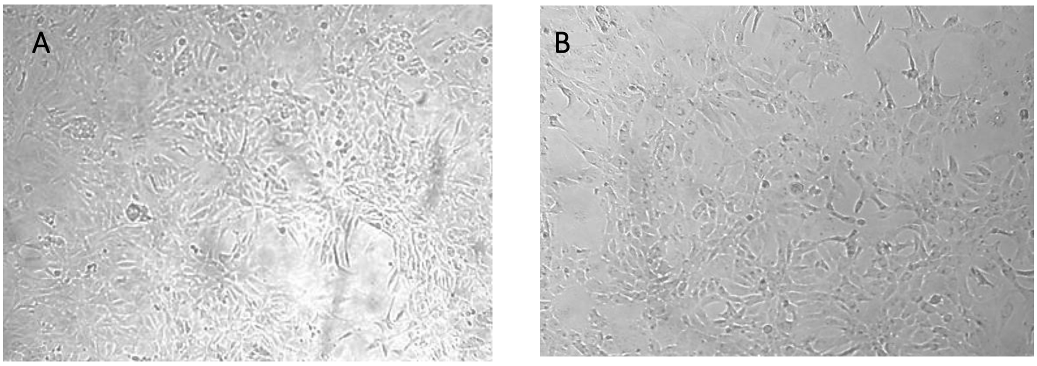

Figure S2 Morphology of NIH 3T3 fibroblasts after 24-hour incubation with **A)** sterile gauze (control) and **B)** with acellular dermal matrix (ADM) without antibacterial agents.

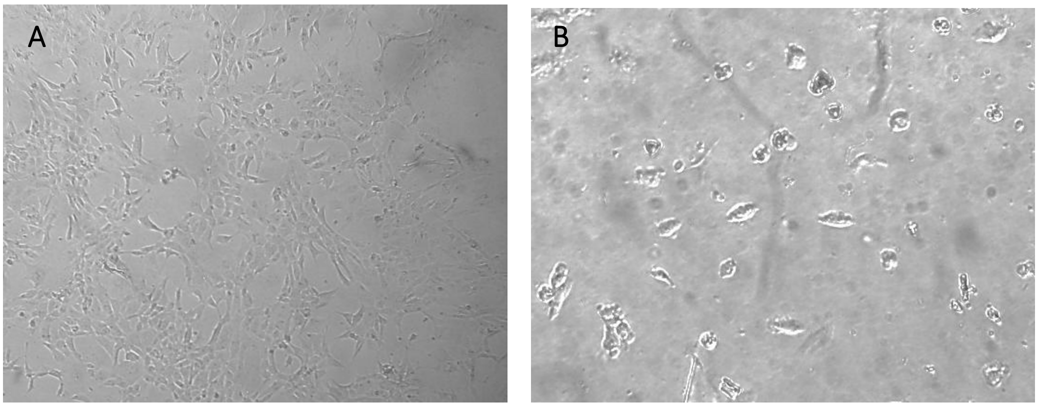

Figure S3 Morphology of NIH 3T3 fibroblasts after 24-hour incubation with 1% acetic acid (**A**) and with Betadine® (**B**).
